# Improved DC Dielectric Performance of cPP-g-MAH/iPP/SEBS Composite with Chemical Graft Modification

**DOI:** 10.3390/ma12071094

**Published:** 2019-04-02

**Authors:** Yu Zhou, Jiaming Yang, Hong Zhao, Weifeng Sun, Mingze Gao, Xindong Zhao, Ming Hu, Shuhong Xie

**Affiliations:** 1 Key Laboratory of Engineering Dielectric and its Application, Ministry of Education, Harbin University of Science and Technology, Harbin 150080, China; 18346181812@163.com (Y.Z.); sunweifenghit@126.com (W.S.); gaomingze00@163.com (M.G.); hlgzxd@163.com (X.Z.); 2 Zhongtian Technology Submarine Cable Co., Ltd., Nantong 226000, China; hum@chinaztt.com (M.H.); xiesh@chinaztt.com (S.X.)

**Keywords:** polypropylene, maleic anhydride, graft, styrene–butadiene–styrene, space charge

## Abstract

In order to achieve both high toughness and favorable dielectric properties of polypropylene materials, a styrene–butadiene–styrene block copolymer (SEBS) was employed as a toughening filler, in addition to a copolymerized polypropylene grafted by maleic anhydride (cPP-*g*-MAH) as a compatibilization modifier, to develop a novel isotactic polypropylene (iPP) composite (cPP-*g*-MAH/iPP/SEBS composite) with significantly improved direct-current (DC) dielectric performance and tenacity. The underlying physical and chemical mechanisms of modifying electric insulation were studied utilizing micro-structure characterization methods in combination with multiple thermal–mechanic–electric tests. The SEBS phase islands are uniformly distributed in the PP matrix with evidently improved dispersion due to cPP-*g*-MAH compatibilization. Compared with iPP, the elastic modulus of cPP-*g*-MAH/iPP/SEBS composites can be reduced by 58% with doubled thermal elongation, which is still superior to that of cross-linked polyethylene (XLPE), implying that the composites are qualified in terms of mechanical properties for use as power cables. The space charge accumulation and electric conduction are considerably suppressed in comparison with pure iPP and the iPP/SEBS composite. In the interest of charge-trapping characteristics modified by chemically grafting MAH, the deep traps introduced into polypropylene by grafting MAH were measured with a thermal stimulation current experiment to be 1.2 and 1.6 eV of energy level in trapping depth, verified through the first-principles electronic structure calculations with an all-electron numerical orbital scheme. It was concluded that the acquired high density of deep traps can effectively restrict the carrier transport and suppress the injection of space charge, resulting in a remarkable improvement of DC dielectric properties for the MAH grafted composites. The present work demonstrates that the cPP-*g*-MAH/iPP/SEBS composites are eligible to be applied to polypropylene-based high-voltage DC cables due to their excellent DC insulation performance, together with the appropriate mechanical properties.

## 1. Introduction

Beyond the traditional alternating-current (AC) transmission system, the flexible direct-current (DC) transmission technology has the advantages of high controllability and a small footprint without cascading fault risk [1,2]. The international power grid advocates the flexible transmission, exploiting high-voltage DC (HVDC) plastic cables [3]. Currently, primary research focuses on the various techniques to modify the insulation materials of cross-linked polyethylene (XLPE) applied in DC plastic cable production, which cannot be recycled due to its thermosetting attributes, thus resulting in severe environmental pollution. Therefore, the exploration of novel insulating materials in virtue of cyclic utilization for green environmental protection is desired [4,5].

Due to the fact that polypropylene (PP) possesses high mechanical strength and excellent insulation performance with favorable processability and recyclability, and that it can operate for a long time at 100 °C, it is expected to develop promising DC cable insulation materials based on PP [6,7]. However, PP cannot be practically used in cable manufacture owing to its high elastic modulus and poor impact strength [8,9,10]. The elastomer was verified as an effective modifying additive to be used for improving the impact strength of PP at low temperature [11,12,13,14,15]. In addition to the mechanical defects, the accumulation of space charge in the polymer insulating material of DC cable limits its applications, which will lead to the distortion of the local electric field, thereby accelerating the electric aging and even causing electric breakdown [16,17,18,19]. At present, a variety of modification techniques were initiated to suppress space charge accumulation in polymer dielectrics, such as propylene–ethylene copolymer blending [20,21], nano-doping [22,23,24,25,26], and graft modification methods [27,28]. He et al. found that the maleic anhydride (MAH) polar groups grafted to PP molecules substantially suppressed space charge accumulation and, thus, increased the DC breakdown field strength and reduced the conductivity of PP grafted with MAH (PP-*g*-MAH) [29]. Zha et al. reported that grafting PP with 2 wt.% MAH could effectively suppress space charge accumulation at room temperature and remarkably decrease conductivity at 70 °C [30]. Zha and He’s studies suggest that grafted MAH can introduce deep traps in PP to improve electrical properties. However, when applying thermally stimulated depolarization current (TSDC) to analyze the trap characteristics, the highest test temperatures for Zha and He’s TSDC tests were only 70 and 100 °C, respectively [29,30]. The melting point of PP is 165 °C, which means that the operating temperature of PP is expected to reach above 100 °C. Therefore, it is necessary to increase the test temperature of TSDC to 165 °C to obtain more comprehensive trap characteristics and to try discover deeper trap levels. In addition, the formation mechanism of deep traps in PP-MAH is still not understood. It is helpful to understand the trap formation mechanism by calculating the local state energy level distribution of PP-MAH via molecular simulation. To achieve appropriate PP-based materials for use in flexible cables, an understanding of whether and how to implement significantly improved dielectric properties through grafting polar molecules such as MAH after necessary mechanical modification by adding toughening fillers such as SEBS is required.

In summary, SEBS as a toughening filler and cPP-*g*-MAH as a modifier for improving the dielectric compatible properties were utilized to develop a cPP-*g*-MAH/iPP/SEBS composite with both high impact strength and dielectric performance. The TSDC characteristics of the materials were tested from the glass transition temperature to the melting temperature, and the mechanism of the formation of deep traps in PP was analyzed via molecular simulation. The physical and chemical properties and DC dielectric performance of the prepared composites were investigated to demonstrate the feasibility of applying graft-modified composites to HVDC insulation materials for plastic cable fabrication.

## 2. Materials and Methods

### 2.1. Material Preparation

The materials chosen in the preparation of modified composites included homopolymer polypropylene (iPP, Sinopec, Beijing, China), SEBS (G1652, Kraton, Houston, TX, USA), copolymerized polypropylene (cPP, FC9413L, TPC, Singapore), antioxidant 1010 (Dongguan Shanyi Plasticizing, Dongguan, China), maleic anhydride (MAH, Ruierfeng Chemical Industry, Quzhou, China), and dicumyl peroxide (DCP, Nobel Company Ltd., Aksu, China). The melt grafting method was adopted in cPP-*g*-MAH preparation as follows: cPP was firstly added into a torque rheometer (RM-200C, Harp Electric Company, Harbin, China) with the temperature set to 190 °C for 3 min; then, the initiators, 3% MAH and 0.2% DCP (mass fraction), were added and mixed for 5 min; finally, the obtained mixture was taken out to be pressed into 0.3-mm-thick flat pieces, with the necessary treatment of removing the ungrafted MAH and oligomer by degassing at 100 °C for 48 h in a vacuum oven. The cPP-*g*-MAH/iPP/SEBS composite was then blended. The cPP-*g*-MAH, iPP, and SEBS in various mass fractions (as listed in the Table 1) for the blend contents of different composites were mixed in a torque rheometer at 190 °C for 3 min with a rotation speed of 40 rpm, after which 0.3 wt.% antioxidant was further added to be mixed for 5 min, eventually procuring composites. It is noteworthy to indicate that the melting press technique was employed in film sample preparation with a 20-min melting process at a temperature of 190 °C and a pressure of 15 MPa, followed by cooling down to ambient temperature at a rate of 5 °C/min.

### 2.2. Characterization and Testing Scheme

Fourier-transform infrared spectroscopy (FTIR, Shimadzu, Kyoto, Japan) was used to characterize cPP and cPP-*g*-MAH samples with a thickness of 0.3 mm in the wave number range from 500–4000 cm^−1^ with a scanning accuracy of 2 cm^−1^. Scanning electron microscopy (SEM, Hitachi, Tokyo, Japan) was implemented to the gold-sprayed samples 0.1 mm in size, which were fractured in a liquid nitrogen environment and etched with *n*-heptane for 20 min. The microstructural morphology of the cross-section was observed with a scanning electron microscope SU8020.

A differential scanning calorimeter (DSC, Mettler Toledo Group, Heppenheim, Germany) was adopted to measure the thermal parameters of the prepared iPP, iPP/SEBS, and cPP-*g*-MAH/iPP/SEBS composites. During the testing experiment, about 5–10 mg of material was added into an aluminum crucible to be heated and cooled down at a rate of 10 °C/min. The material melted completely after heating from 30 to 200 °C, and this temperature was maintained for 2 min to eliminate internal stress; then, the thermal spectra of the crystallization process could be measured with the temperature returning to 30 °C, and a further melting process could be tested when the samples were reheated to 200 °C. The crystallinity (*X*_C_) characterizes the proportion of the crystalline content in a material; it can be obtained using the following calculation formula from the DSC thermal spectra:

(1) $$X_{c}=\frac{\Delta H_{m}}{\Delta H_{100}}\times 100\%$$

where ∆*H*_m_ and ∆*H*_100_ (J/g) denote the melting temperatures of the composite and pure crystal with 100% crystallinity, respectively.

Thermal elongation tests were performed to investigate the mechanical properties at high temperature, in which the sample with a thickness of 1 mm and a dumbbell shape with a middle width of 4 mm was placed in a baking oven, with the other side loaded with a counterweight; this process was carried out for 15 min at the set temperature so as to eventually measure the elongations. Specially, dynamic mechanical analyses (DMA) were implemented with a Dynamic Thermomechanical Analyzer Q800 (TA Instruments Company, New Castle, DE, USA) to effectively evaluate whether the insulation material could maintain the necessary mechanical strength at the desired overload temperature, in which the samples were cuboidal with dimensions of 1 × 4 × 9 mm^3^, and tested at a 1-Hz frequency at temperatures increasing from −80 °C to 160 °C at a heating rate of 3 °C/min.

A three-electrode system was employed to measure the conductivity of the 0.2-mm-thick samples. A high-voltage aluminum electrode (76 mm in diameter) was vaporized, along with an aluminum guard electrode (54 mm inner diameter and 74 mm outer diameter) and an aluminum measuring electrode (50 mm in diameter). Quasi steady-state current was measured using a Keithley 6517A (Cleveland, OH, USA) at individual temperatures of 30, 50, 70, and 90 °C under 3‒40 kV/mm electric fields. The space charge characteristics of iPP, cPP-*g*-MAH/iPP/SEBS, and iPP/SEBS composites were measured via the pulse electroacoustic (PEA) method, whereby aluminum electrodes were deposited onto both surfaces of the specimens via sputtering. The diameter of the aluminum electrode was 25 mm and the thickness of the specimens was 0.3 mm; the samples were measured under a DC electric field of 20‒40 kV/mm for 15 min and short-circuited for 30 min, with all measurements performed at room temperature (25 ± 3 °C). The energy level distribution of charge traps was measured via thermally stimulated depolarization current (TSDC) on the 0.1-mm-thick samples with an applied electric field of 40kV/mm in a vacuum environment at 60 °C for a 30-min polarization, after which the temperature rapidly decreased to −30 °C due to the inlet of liquid nitrogen through a vacuum vessel. Then, the sample was short-circuited to be measured for the depolarization current with the temperature increased from −30 to 155 °C at a heating rate of 3 °C/min.

### 2.3. Theoretical Calculation Methodology

The molecular models for representative PP-*g*-MAH were initially constructed according to the equilibrium C–C and C–H bond lengths with random distributed torsion, in which the PP molecules with a polymerization degree of 60 were are grafted with an MAH molecule near the middle position of the PP backbone chain, based on a rotational isomeric state (RIS) model. The constructed initial polymer conformations were geometrically optimized to relaxation via total energy functional minimization of first-principles calculations such that the energy change, atomic force, and displacement were lower than 1.0 × 10^−5^ eV/atom, 0.03 eV/Å, and 0.001 Å, respectively. The electronic structures were calculated for molecular orbitals and electronic density of sates to investigate band-edge features and grafting-introduced trap states. The first-principles calculations were performed employing the scheme of all-electron and numerical atom orbitals as implemented in the DMol3 program of the Materials Studio 8.0 software package, as detailed in the calculations listed in Table 2. 

## 3. Results and Discussion

### 3.1. Structure Characterization

**Figure 1:**
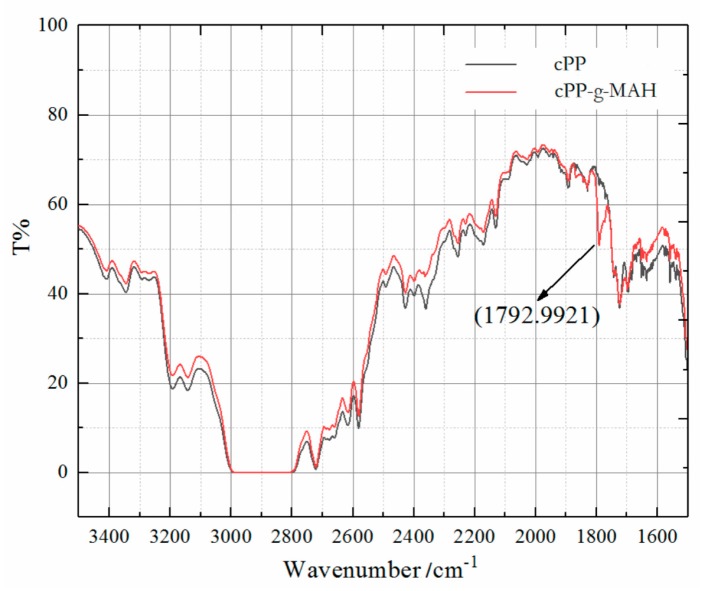
Fourier-transform infrared (FTIR) spectra of copolymerized polypropylene (cPP) and cPP grafted by maleic anhydride (cPP-*g*-MAH).

The polar groups of MAH were chemically grafted to cPP molecules in cPP-*g*-MAH, as verified by the characteristic absorption peaks at 1793 cm^−1^, corresponding to ester C=O stretching vibration, from the IR spectroscopy, which newly appeared in contrast to cPP, as shown in Figure 1. Meanwhile, the characteristic C=C vibration absorption peak at 1592 cm^−1^ of the MAH monomer disappeared, demonstrating that the remaining ungrafted MAH molecules were completely degassed from the cPP-*g*-MAH.

**Figure 2:**
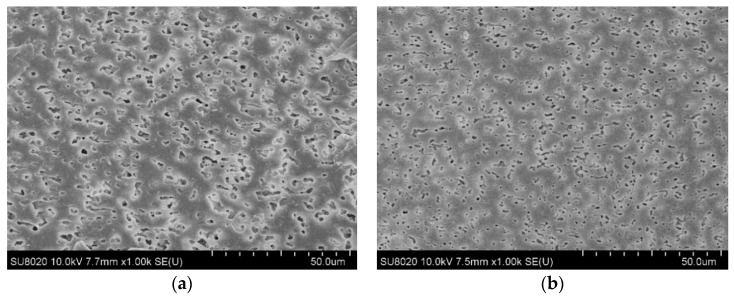
SEM images of (**a**) isotactic polypropylene (iPP)/styrene–butadiene–styrene block copolymer (SEBS), and (**b**) cPP-*g*-MAH/iPP/SEBS.

The SEM images of the two composites are shown in Figure 2. The voids in the SEM images are due to the etched SEBS phase. PP is a non-polar material, while SEBS has a block copolymerized polystyrene unit, which has a certain polarity [31]. It can be seen from Figure 2a that PP and SEBS cannot be blended homogeneously, and SEBS distributes as an island structure in the PP matrix. It can be further noted from Figure 2b that the size of the dispersed SEBS phase decreases, and the degree of dispersion improves obviously. It shows that cPP-*g*-MAH increases the polarity of the PP phase, contributes to the interfacial bonding of various phases in the composites, and significantly improves the compatibility of the PP and SEBS phases.

### 3.2. Thermal–Mechanical Performance

**Figure 3:**
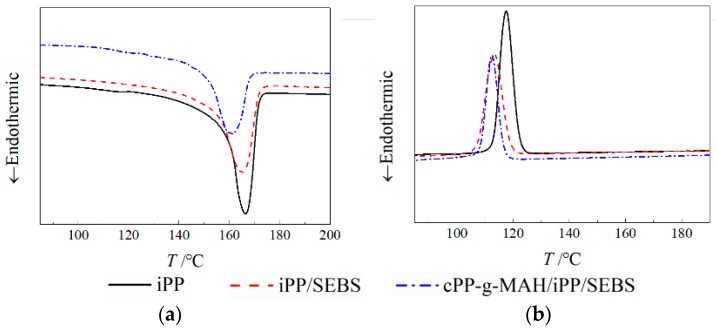
Differential scanning calorimetry (DSC) temperature spectra in melting (**a**) and crystallization (**b**) processes for different samples.

The crystallization temperature *T*_c_ and melting temperature *T*_m_ for iPP/SEBS were both lower than that of iPP, as exhibited in Figure 3, which presents the DSC characteristic spectra of iPP and the two composites, implying that the SEBS phase with dispersed island structures hinders the growth of lamellar crystals in the iPP crystallization process, resulting in decreased *X*_C_ and imperfect crystallization morphology. It is indicated from the discrete values listed in Table 3 that the Tm and *X*_C_ of the cPP-*g*-MAH/iPP/SEBS composite are further reduced in comparison with iPP/SEBS. The complex branched-chain structure of cPP-*g*-MAH with low symmetry led to large steric hindrance for the substitution group and hindered crystallization. Meanwhile, the addition of cPP-*g*-MAH improved the compatibility between SEBS and PP by increasing the interaction between their phase interfaces, resulting in higher dispersivity. Moreover, the collaborative effect of SEBS with cPP-*g*-MAH suppressed the crystallization process of the PP phase, and consequently led to further decreased crystallinity. The results of the elongation test are shown in Table 4. Compared with iPP, the deformation of the two composites increased at 155–165 °C, which is attributed to the island-distributed SEBS with high flexibility. The results of the elongation test show that the mechanical properties of the composite are excellent at high temperature, and the elongation of the composite is still high when the rigidity decreases.

**Figure 4:**
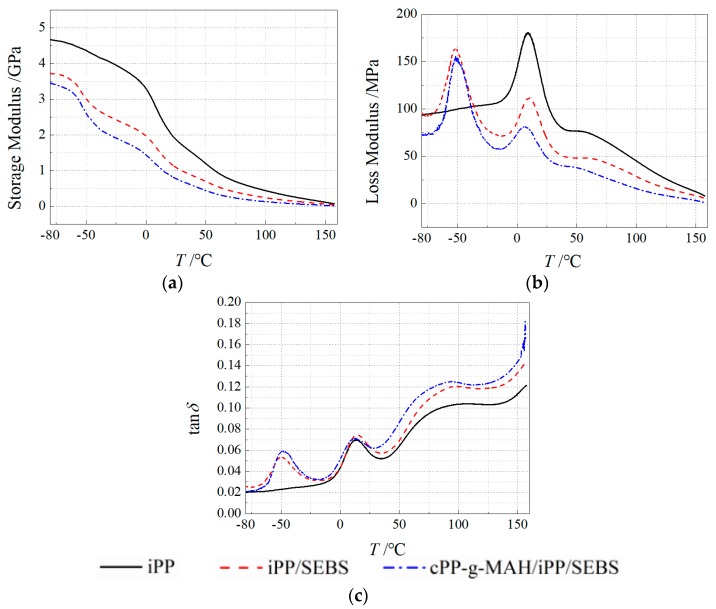
The dynamic mechanical analysis (DMA) thermal spectra of (**a**) storage modulus, (**b**) loss modulus, and (**c**) tanδ for iPP and its composites.

The temperature-varying spectra of storage modulus (*E*′), loss modulus (*E*″), and loss factor (tan*δ*) for iPP and composites were obtained from the DMA tests, as shown in Figure 4. The *E*′ values of the two composites were obviously lower than that of iPP. At 25 °C, the *E*′ values of iPP/SEBS and cPP-*g*-MAH/iPP/SEBS composites were remarkably reduced to 1100 MPa, compared with the 1800 MPa of iPP. These results are attributed to the intrinsic low elastic modulus of SEBS and they are correlated with the impeded crystallization of PP from the enhanced compatibility between SEBS and PP.

The relaxation loss peaks appearing at −50 °C and 20 °C, as shown in Figure 4c, were caused by the glass transitions of the SEBS phase and iPP phase [14,31]. Compared with iPP/SEBS, the loss peak at −50 °C of the cPP-*g*-MAH/iPP/SEBS composite moved slightly to a higher temperature and the peak intensity increased, while the loss peak at 20 °C shifted to a lower temperature with a smaller peak value. The loss peak at −50 °C was primarily derived from the relaxation process of the EB chain segment in SEBS. The polar groups in cPP-*g*-MAH restrain the movement of EB chain segment and, thus, make the loss peak shift to a higher temperature. The polar group of cPP-*g*-MAH also improves the SEBS dispersion in PP, which reduces the size of the dispersed SEBS phase and increases the interface areas and the internal friction of chain segment movement, thereby leading to the loss peak intensity. The loss factor at 20 °C originated mainly from the chain relaxation in the amorphous region of the PP phase. The dispersion of SEBS improved and the crystallization process of PP phase was inhibited when cPP-*g*-MAH was filled, such that the chain segments could be more easily relaxed in the amorphous region, leading to reduced temperature and intensity of loss peak. The loss factor peak around 80 °C corresponds to the α-relaxation process in the material, which represents the relaxation behavior of structural defects in the crystal region. Compared with the loss peaks of the three materials, it is noted that the loss peaks shifted to lower temperature after SEBS was filled, and relaxation temperature further decreased following cPP-*g*-MAH modification. It is suggested that the SEBS leads to the easier relaxation of chain segments at the interface between PP and SEBS phases, and the cPP-*g*-MAH can further intensify this effect by improving the dispersion of SEBS. It is also found from the results of Figure 4c that the tan *δ* peak intensities of modified composites were higher than that of iPP, which was mainly attributed to the partial chain segments of SEBS entering the PP phase and increasing the friction force in the relaxation movement. Furthermore, the lamellar crystals of PP grow to the interface area between SEBS and PP phases so as to generate more structural defects, resulting in a higher intensity of the tan δ peak. The variation of the energy loss in relaxation processes depends on the interface area between SEBS and PP phases, which is determined by the specific area of SEBS; this explains why the tan δ of the cPP-*g*-MAH/iPP/SEBS composite was relatively larger.

### 3.3. Electrical Conductivity

**Figure 5:**
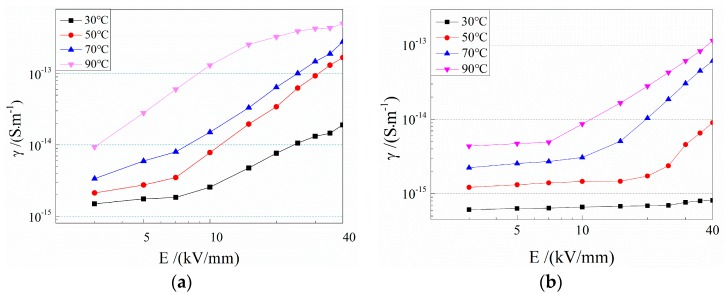
The electrical conductivity of (**a**) iPP/SEBS and (**b**) cPP-*g*-MAH/iPP/SEBS, as a function of electric field at 30–90 °C.

The electrical conductivities of the two composites varying with applied electric field at different temperatures are shown in Figure 5, illustrating the obviously lower conductivity of the cPP-*g*-MAH/iPP/SEBS composite compared to that of the iPP/SEBS composite. It is also found that the conductivity of iPP/SEBS begins rising with increasing electric field when the electric field exceeds 7 kV/mm at room temperature, and the current density no longer maintains a linear relationship (Ohmic law) with the electric field. In contrast, the cPP-*g*-MAH/iPP/SEBS composite exhibits almost constant electrical conductivity within the measured range of electric field. The nonlinear thresholds of electric field for both composites decreased gradually with the increase in testing temperature, with a higher value of the cPP-*g*-MAH/iPP/SEBS composite than that of the iPP/SEBS composite.

The conductivity of the material is proportional to the charge carrier concentration and mobility. The carrier in the tested polymer insulating materials mainly came from electrode injection and impurity ionization. Based on Schottky and Poole–Frankel effects, the nonlinear relationship between conductivity and electric field implies that the increment of electric field can increase the carrier concentration and reduce the barriers of carrier transport, which results in increased carrier mobility. On the other hand, the increment of temperature intensifies the thermal motion of carriers, causing a decline in the nonlinear threshold electric field of conductivity [32].

It was reported that the polar groups of MAH grafted to PP can introduce uniformly distributed deep traps at high density, which can effectively decrease carrier migration, as verified here by the lower conductivity and higher nonlinear threshold electric field of the cPP-*g*-MAH/iPP/SEBS composite than that of the iPP/SEBS composite [29,30]. In addition, the conductivities of the two composites approached the same values with increasing temperature, indicating that the effect of deep traps derived from grafted MAH leading to the suppression of carrier generation and migration is weakened at higher temperatures. Accordingly, it is suggested that the conductivity reduction depends on the depth of charge traps introduced by MAH, and they lose efficacy upon thermal excitation of the trapped charges at adequately high temperature. 

### 3.4. Space Charge Characteristics

**Figure 6:**
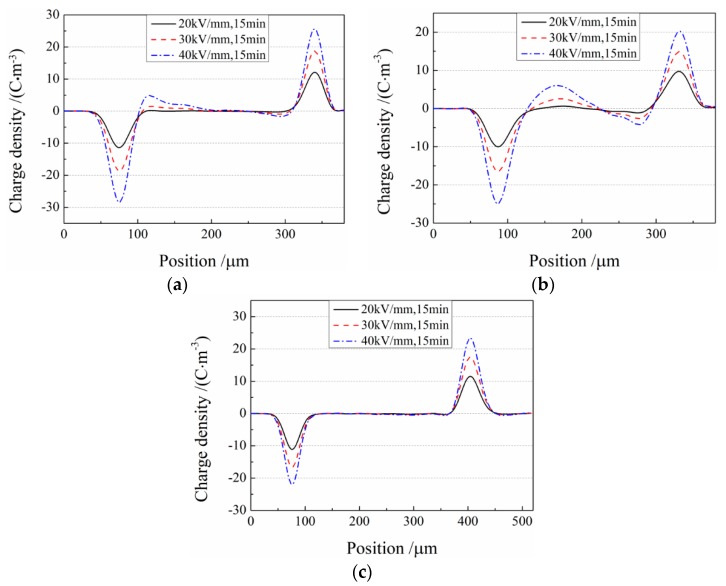
Space charge distributions of (**a**) iPP, (**b**) iPP/SEBS, and (**c**) cPP-*g*-MAH/iPP/SEBS after polarization by the direct-current (DC) electric field of 20, 30, and 40 kV/mm for 15 min.

As shown in Figure 6, demonstrating the space charge distributions of iPP, iPP/SEBS, and cPP-*g*-MAH/iPP/SEBS composites under applied electric fields of 20, 30, and 40 kV/mm for 15 min at ambient temperature, iPP and iPP/SEBS samples exhibited heterocharges accumulated near the cathode and anode, and the density of space charge accumulated in the iPP/SEBS composite was obviously higher than that in iPP. With the increase in electric field, the heterocharge in iPP and iPP/SEBS composites accumulated gradually and migrated inside the samples, which was not represented for the cPP-*g*-MAH/iPP/SEBS composite. Under the applied electric field, the positive and negative charges caused by electrode injection and ionization of impurities propogated in opposite directions and eventually accumulated near electrodes to form the heterocharge [7,17]. It is interestingly noted that the space charge accumulation greatly intensified with the addition of 25% SEBS (as for the iPP/SEBS composite), while it was obviously suppressed by the addition of cPP-*g*-MAH (as for the cPP-*g*-MAH/iPP/SEBS composite). The charge is more likely to migrate in the amorphous SEBS phase than in the crystalline PP; thus, the charge can transport through SEBS islands into the interior of the iPP/SEBS composite, before being captured by traps in the PP phase so as to form more space charges. It was reported that grafting MAH to PP can effectively suppress space charge accumulation due to the introduction of deep traps in PP by the polar group of MAH [33,34]. It should be emphasized here that the deep trap must have a high enough density to engender the suppression effect for which the injected charges are immediately captured and restricted to the electrode, and for which the internal charges produced by impurity ionization cannot migrate toward the electrodes. After mixing the cPP-*g*-MAH, the grafted MAH uniformly dispersed at a high density generates an effective charge-screening layer on the material surface contacted by the electrodes, thereby capturing charge carriers injected from the electrodes and suppressing further space charge injection.

**Figure 7:**
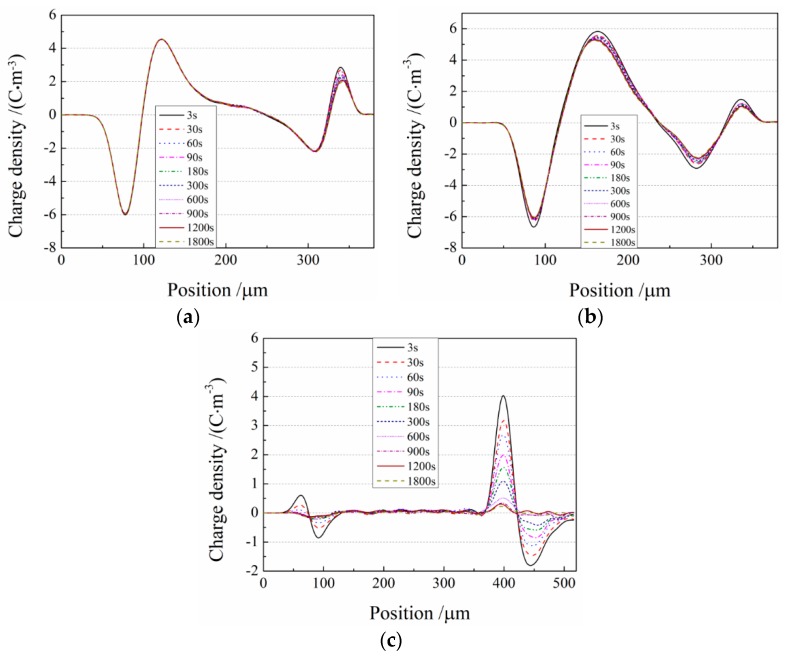
Space charge distributions during depolarization for (**a**) iPP, (**b**) iPP/SEBS, and (**c**) cPP-*g*-MAH/iPP/SEBS.

The space charge distributions in the short-circuit process of the three materials are shown in Figure 7. After short circuit, the accumulated space charges in the cPP-*g*-MAH/iPP/SEBS composite were almost completely released, while the space charges in the iPP and iPP/SEBS samples decayed slowly. After 30 min, the accumulated space charge density in the iPP, iPP/SEBS, and cPP-*g*-MAH/iPP/SEBS samples approached 4.5 C/m^3^, 5.3 C/m^3^, and less than 0.5 C/m^3^, respectively, due to the remarkably faster charge decay in the cPP-*g*-MAH/iPP/SEBS composite. The average bulk charge density at different times ρ(*t*) during the short-circuit process is calculated using the following formula:

(2) $$\rho (t)=\frac{1}{x_{1}-x_{2}}\int_{x_{1}}^{x_{2}}\left|\rho (x,t,E_{p})\right|dx$$

where *x*_1_ and *x*_2_ signify transverse coordinates of the cathode and anode, respectively, and ρ(*t*,*x*,*E*_p_) identifies the charge density at the *x* position of the internal sample under a polarization field at time *t* [35]. 

**Figure 8:**
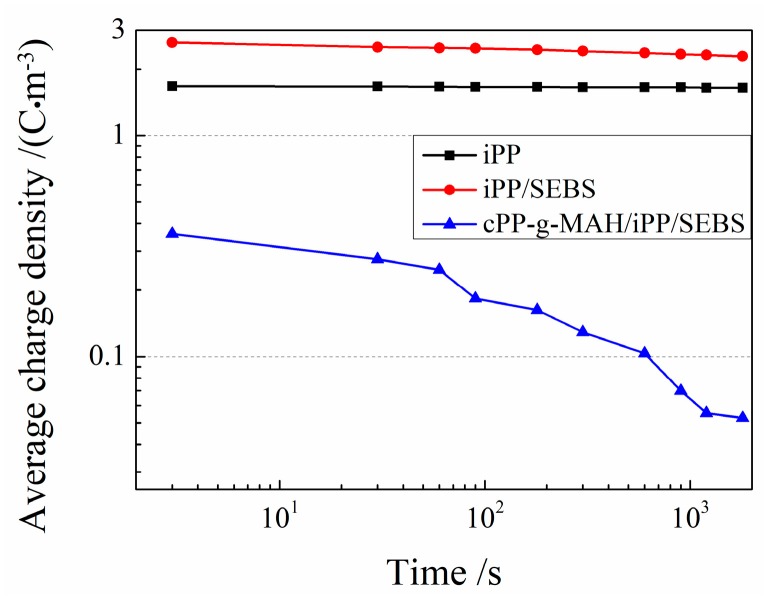
The average space charge density varying with time during the depolarization process.

The average densities of space charges for the three investigated materials in the depolarization process were calculated using Equation (2), with the results shown in Figure 8, confirming the lowest space charge accumulated in the cPP-*g*-MAH/iPP/SEBS composite due to the shallow traps introduced by the structural defects at the interfaces between cPP-*g*-MAH and SEBS phases.

### 3.5. Charge Trap Characteristics

**Figure 9:**
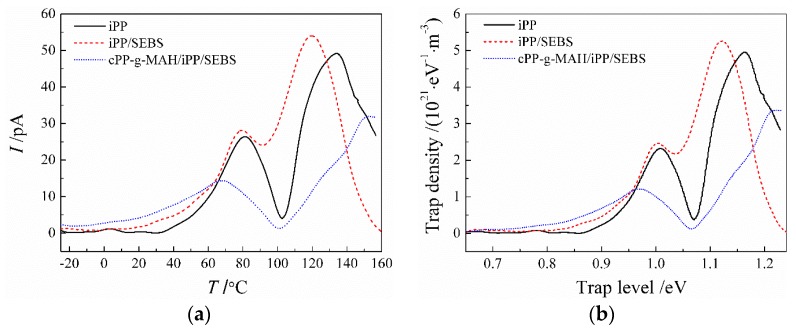
(**a**) Temperature spectra of thermally stimulated depolarization current (TSDC), and (**b**) the derived trap level distributions.

The TSDC spectra of iPP, iPP/SEBS, and cPP-*g*-MAH/iPP/SEBS (Figure 9) illustrate the two characteristic peaks of current release at 80 and 135 °C from the iPP matrix. It was reported by Ieda that the similarity of the TSDC spectrum and the DMA loss spectrum reasonably implies the correlation of charge traps to structural defects [32]. Comparing the results presented in Figure 9 and Figure 4b, it is confirmed that the TSDC spectra of iPP and iPP/SEBS are similar to the DMA loss spectra. From the previous analysis of tanδ spectra for iPP, corresponding to the α-relaxation process of iPP at 80 °C, the thermal current comes from the release of trapped charge caused by structural defects between crystalline regions. It is also indicated from the DSC spectra shown in Figure 3 that the endothermic reaction appreciably increases at the initial temperature of 135 °C, corresponding to the point at which the TSDC peak mainly originates from the charge release during the melting process of crystal regions.

In comparison to iPP, the two current peaks of the iPP/SEBS composite shift to a lower temperature and increase in intensity. It was verified by the lower crystallizing and melting temperatures from previous DSC tests that SEBS depresses the iPP crystallization process and results in convenient relaxation of the PP molecular chain, which accounts for the lower temperature of TSDC peaks for the iPP/SEBS composite. Structural defects produced in the incomplete crystallizing process introduce charge traps, which can be arguably identified by the increased DMA tan*δ* and TSDC peak intensity. In contrast, the low-temperature current peak of the cPP-*g*-MAH/iPP/SEBS composite appears at 65 °C, which is significantly lower than that of iPP and iPP/SEBS, while the currents in the range from −20 to 65 °C are higher. The results show that the number of shallow traps in cPP-*g*-MAH/iPP/SEBS increases, which is attributed to the improved compatibility of SEBS and the further destroyed crystallization of iPP. In the high-temperature region, the current release peaks appearing in iPP and iPP/SEBS are obviously suppressed and shift to the higher temperature of 155 °C as for the cPP-*g*-MAH/iPP/SEBS composite. As indicated in the DSC and DMA tests, the crystallization process of iPP was further destroyed by the addition of cPP-*g*-MAH. Therefore, the current release peak at high temperature was not contributed from traps on structural defects in the iPP crystal region, but from deep traps introduced by the grafted MAH. Furthermore, the space charge decay in the short-circuit experiments shows that the numbers of both deep and shallow traps in cPP-*g*-MAH/iPP/SEBS increase, which is confirmed by the TSDC tests and is consistent with DMA and DSC tests. It should be emphasized that the increments of shallow and deep traps are due to a further suppression of crystallization and by grafting MAH, respectively. 

**Figure 10:**
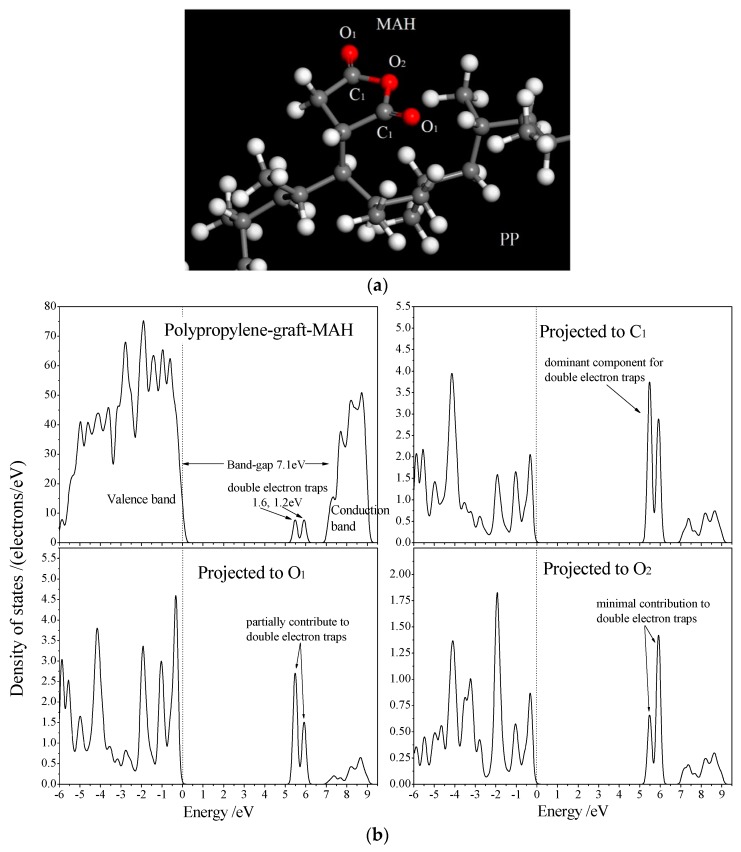
(**a**) Schematics of grafting MAH to polypropylene with different oxygen atoms being identified by O_1_, O_2_, and C_1_; (**b**) the total and O-projected density of states for PP-graft-MAH from first-principles calculations.

According to the method presented by Reference [36], TSDC spectra can be converted to energy spectra (reference to trapping level depth) of trap density as shown in Figure 9b. The energy level depth of deep traps in cPP-*g*-MAH/iPP/SEBS was calculated to be about 1.2 eV, which agrees well with the density of state (DOS) results from the first-principles electronic structure calculations, as shown in Figure 10 for the relaxed structure following geometry optimization and the total/projected DOS of PP grafted with MAH (PP-*g*-MAH).

MAH grafted to PP molecules introduces two unoccupied bound states in the band-gap of PP molecules, which act as the deep traps of conducting electrons with energy level depths of 1.2 eV and 1.6 eV. Next to the conduction band minimum of PP, a lower-level bound state is introduced into the conduction band to form a new band edge, resulting in a 0.8-eV reduction of the band gap. The projected density of states (Figure 10b) shows that the two trap states are mainly derived from the carbonyl carbon atom (C_1_) and a small fraction from the carbonyl oxygen atom (O_1_) of the grafted MAH, where the electron wave functions are distributed in local space around the carbonyl bond (C=O). Two adjacent C=O bonds in grafted MAH overlap and result in two unoccupied splitting molecular orbitals, which can capture electrons in the conduction band of PP as electron-bound states. The trap states of 1.6 eV are composed of 60% C_1_ and 39% O_1_ atomic orbitals, while the traps of 1.2 eV mainly come from C_1_ atomic orbitals (>70%) with a minor part (<30%) from atomic orbitals of O_1_ and O_2_ (ester oxygen). The electronic structure results of theoretical calculations are consistent with the experimental results of TSDC, further proving that the anhydride group in grafted polar molecules can introduce electronic states of deep traps with energy levels amongst the band gap of PP, and the coupling of anhydride groups in grafted molecule (such as MAH) produces a pair of deep traps at high density in the conduction band of PP. The mechanism underlying deep traps suppressing space charge involves them effectively capturing carriers before blocking the charge emission of the electrode and suppressing the charge migration to the material [25,33]. The condition necessary to ensure the existence of the deep trap mechanism requires that the charge captured by the trap is not released because of the increase of temperature; thus, cPP-*g*-MAH/iPP/SEBS with deeper trap levels means that it has a space charge suppression effect at higher temperature, which also means that it can operate at higher temperature. 

## 4. Conclusions

The cPP-*g*-MAH/iPP/SEBS composite was developed with chemical grafting and polymer mixing methods and investigated through microstructure characterizations, as well as thermal–mechanical and dielectric performance tests. The compatibility of SEBS islands with the PP matrix was significantly increased by filling cPP-*g*-MAH, and the thermal elongation at 165 °C was only 20%, even though the crystallinity and melting temperature were reduced. The low conductivity under a high electric field at 90 °C and the evidently suppressed space charge accumulation at ambient temperature in cPP-*g*-MAH/iPP/SEBS demonstrate the excellent DC dielectric performance acquired by grafting MAH. The consistent results of the first-principles electronic structure calculations with the TSDC tests suggest that the electron deep traps of 1.2 eV and 1.6 eV in energetic depth can be introduced by the anhydride group of the grafted molecule into the PP matrix, in which the pair of deep traps at high density is derived from the coupling of anhydride groups (such as MAH) and results in strong scatter and the capture of conduction-band electrons. 

## Figures and Tables

**Table 1:** Blend contents for preparing composite samples (wt.%). cPP-*g*-MAH—copolymerized polypropylene grafted by maleic anhydride; iPP—isotactic polypropylene; SEBS—styrene-butadiene-styrene block copolymer.

Samples	cPP-*g*-MAH	iPP	SEBS
iPP/SEBS	0	75	25
cPP-*g*-MAH/iPP/SEBS	37.5	37.5	25

**Table 2:** Methodology and parameters adopted in first-principles calculations.

Electronic Hamiltonian	Scheme	Condition and Parameter
Exchange–correlation energy	Meta-generalized-gradient approximation	Multi-configurational dual-range local exchange (M11-L)
Integration accuracy	–	2000 grid points /atom
Self-consistent field	Tolerance	1 × 10^−6^ eV/atom
	Multipolar expansion	Octupole
	Charge density mixing	Charge = 0.3, Direct inversion iterative subspace (DIIS) = 5
Core treatment	All-electron	–
Numerical basis set	Double numerical polarized	Basis file 4.4
Orbital cutoff	Global	5.0 Å

**Table 3:** Thermal parameters from differential scanning calorimetry (DSC) test for different samples.

Sample	iPP	iPP/SEBS	cPP-*g*-MAH/iPP/SEBS
*T*_c_ (°C)	117.5	113.7	111.9
*T*_m_ (°C)	166.5	165.4	162.2
*X*_C_ (%)	39.91	35.65	28.23

**Table 4:** Thermal elongation of different samples at 155 and 165 °C (%).

Samples	iPP	iPP/SEBS	cPP-*g*-MAH/iPP/SEBS
155 °C	5	9	10
165 °C	7	17	20
